# Amelogenesis Imperfecta: A Case Series from the Community

**DOI:** 10.31729/jnma.3709

**Published:** 2018-12-31

**Authors:** Abanish Singh, Santosh Kumari Agrawal, Ashish Shrestha, Tarakant Bhagat

**Affiliations:** 1 Department of Public Health Dentistry, BP Koirala Institute of Health Sciences, Dharan, Nepal

**Keywords:** *Amelogenesis Imperfecta*, *esthetic*, *yellowish discoloration*

## Abstract

Amelogenesis Imperfecta is a hereditary disorder affecting the formation of enamel structure. Two female children and one male (11 years, 12 years and 6 years respectively) reported with chief complaint of yellowish discoloration of teeth since their childhood. They reported that they had similar discoloration in their deciduous teeth. Clinical examination showed generalized deposits of plaque and calculus, yellowish discoloration of the teeth with chipping off of the incisal and cuspal enamel structures. OPG revealed thin lining of enamel with thick dentin layer and pulp chamber. PA view revealed unfused anterior fontanels and lateral cephalogram indicated vertebrae in growing phase. The patients were instructed to maintain proper oral hygiene and regular follow up till the growth cessation. Permanent skeletal, functional, esthetic needs is addressed after growth completion. Oral rehabilitation through multidisciplinary approach can certainly provide a good prognosis and patients were counselled and motivated to maintain good oral hygiene.

## INTRODUCTION

Amelogenesis Imperfecta (AI) is a hereditary disorder affecting the formation of enamel structure in both primary and permanent dentition.^1^ These disorders have compromised enamel qualitatively and quantitatively resulting in compromised function and esthetics.^2^ In 1976, Witkop and Sauk classified based on whether the abnormality lie in reduced amount of enamel (hypoplasia), deficient calcification (hypocalcification), imperfect maturation of the enamel (hypo-maturation), or combined defects.^3^ Depending upon the gene pool its epidemiology varies from 1:14,000 to 1:700 in the USA.^3,4^ Hypoplastic AI represents 60 to 73%, hypo-maturation AI represents 20 to 40%, and hypocalcification AI represents 7% of all the cases.^5^

## CASE REPORT

### CASE 1

A 12-year-old female reported with chief complaint of yellowish discoloration of teeth since her childhood at a community camp in Mahendranagar, Sunsari. She reported that she had similar discoloration in her deciduous teeth. Examination showed mandibular prognathism, anterior open bite, generalized deposits of plaque and calculus, yellowish discoloration of the teeth with chipping off of the incisal and cuspal enamel structures. Orthopantomogram (OPG) revealed thin lining of enamel with thick dentin layer and pulp chamber. Postero-anterior (PA) view radiograph revealed unfused anterior fontanels and lateral cephalogram indicated vertebrae in growing phase. The patient and her parents were counselled regarding treatment plan and prognosis. Full mouth ultrasonic scaling was performed. As the child was in her growing phase, permanent rehabilitation of the teeth was not recommended. Hence, patient was instructed for maintenance of proper oral hygiene, diet precautions and regular follow-up.

**Figure 1.**
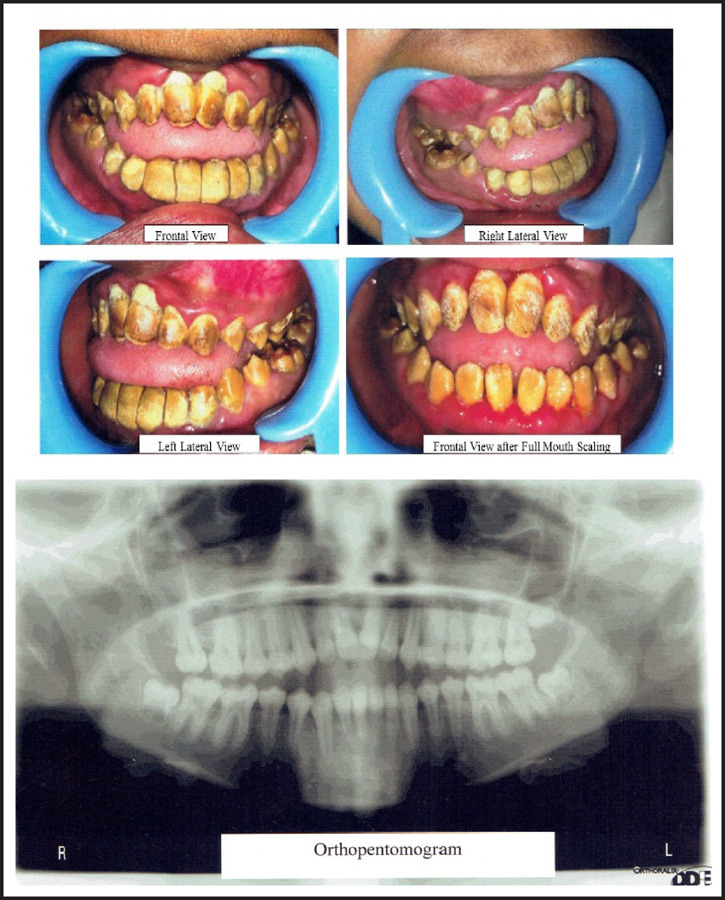
Pictures of Case 1.

### CASE 2

An 11-year-old female reported at community outreach program Hansposa, Sunsari with chief complaint of brown to yellowish discolouration of teeth and sensitivity and difficulty while eating. She had similar discolouration in her deciduous dentition. Similar condition was also reported in her brother indicating hereditary predilection. Examination showed normal facial profile with normal molar occlusion, brown to yellowish discolouration of the teeth with chipping off cuspal enamel in posteriors. OPG revealed thin lining of enamel with thick dentin layer and pulp chamber. The treatment was deferred till the growth cessation and eruption of full set of teeth (permanent).

**Figure 2.**
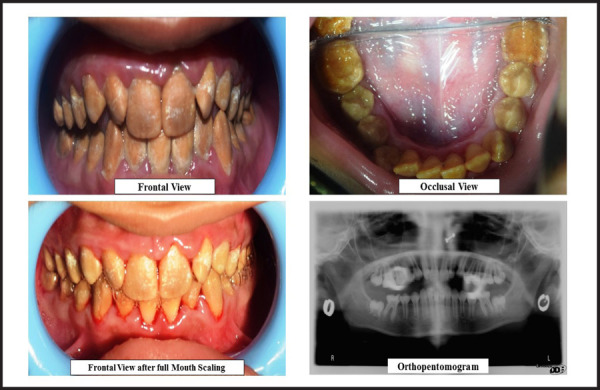
Pictures of Case 2

### CASE 3

A six-year old male child reported at community outreach program Hansposa, Sunsari with chief complaint of sensitive teeth and mobile teeth in front region of lower jaw. Similar condition was reported in his elder sister. Examination showed normal facial profile with normal occlusion, brown to yellowish discolouration of the teeth with chipping of cuspal enamel in posteriors. Exfoliative mobile 71 and 81 with lingually erupting permanent lower central incisors were also yellowish in colour. Patient was referred to the institution for further treatment. Restoration of chipped off 55 and 65 was done with GIC and extraction of 71 and 81 was done under local anaesthesia. Patient counselling was done and was asked to have regular follow-ups till all the permanent teeth erupt.

**Figure 3.**
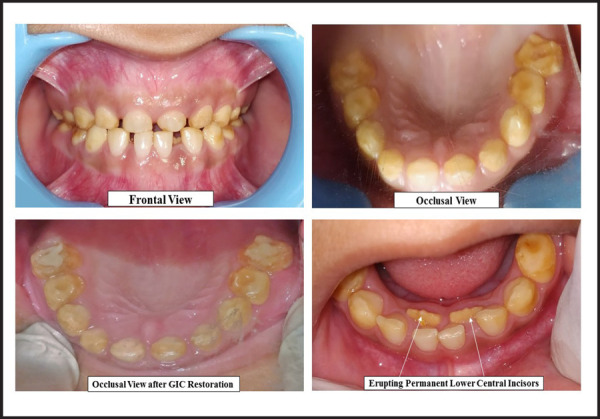
Pictures of Case 3.

## DISCUSSION

Amelogenesis Imperfecta is a hereditary disorder affecting the formation of enamel structure. It results in compromised function and esthetic of a tooth. This disorder shows autosomal dominant, autosomal recessive, sex-linked and sporadic inheritance patterns without systemic manifestation.^1,7^ Diagnosis of AI is made on the basis meticulous recording of case history with family history, pedigree plotting and clinical observation.^7^ Intraoral radiograph showed the relative difference between the radiodensity of the enamel and dentin. The commonest differential diagnosis of AI includes dental fluorosis and chronological enamel hypolasia.^7^

Formation of enamel is highly organized and its formation during organogenesis is controlled through a number of organic matrix molecules like enamelin (ENAM; 4q21), amelogenin (AMELX;Xp22.3-p22.1), ameloblastin (AMBN;4q21), tuftelin (TUFT1;1q21.), amelotin (AMELOTIN 4q13), dentine sialophosphoprotein (DSPP; 4q21.3), and a variety of enzymes, such as kallikrein 4 (KLK4;19q13.3-q13.4) and matrix metalloproteinase 20 (MMP20;11q22.3-q23). Histological ground section of AI shows very thin enamel with laminations of irregular enamel prisms.^8^

Treatment of patients with AI needs addressing of both clinical and emotional demands. Patients with AI are found to be covering teeth with papers and chewing gums to mimic the normal tooth.^8^ Treatment of AI emphasizes more on preventive care rather than curative. The primary dentition is protected by the use of preformed metal crowns on posterior teeth. Either polycarbonate crowns or composite restorations are used on anterior teeth. The permanent dentition requires preformed metal crowns on posterior teeth, as they erupt and composite restorations on anterior teeth. The anterior open bite seen in some cases of AI requires consideration of surgical as well as restorative management.^7,9,10^

There was limitation in providing complete treatment to all the patients as all of them were in their growing stage. The patient also belonged to poor socioeconomic background so that they could not afford the crowns and veneers at current stage. The basic oral prophylaxis and restoration were provided free of cost. Dental and skeletal maturity are yet to be completed after which the final treatment can be done. Oral rehabilitation through multidisciplinary approach can certainly provide a good prognosis and patient should be counselled and motivated to maintain good oral hygiene. Some insurance must be assured by the government level for such underprivileged children with hereditary oral abnormalities.

This case series gives an insight about such rare disease hidden in the community that are being ignored due to lack of education, awareness about treatment options available and financial burden.

## Consent

JNMA Case Report Consent Form was signed by the patient and the original article is attached with the patient's chart.

## Conflict of Interest

**None.**
